# Cost elements to be considered in estimates for microcosting studies in peritoneal dialysis (PD) therapy in Latin America

**DOI:** 10.1590/2175-8239-JBN-2025-0202en

**Published:** 2026-02-09

**Authors:** Celso Souza de Moraes-, Natalia Orihuela, Natália Maria da Silva Fernandes, Laura Cortes Sanabria, Luciane Senra de Souza Braga, Pablo Amair, Uriel Winik, Angelica Viviana Manchinelli, Marta Susana Adragna, Eliseo Antonio Guzmán, Cristina Vallve, Roger Ayala Ferrari, Rodrigo José Álvarez, Hernán Jaramillo Mendoza, Viviane Calice-Silva, Erwin Iván Campos, Raul Plata-Cornejo, Regulo Valdes, Ricardo Silvariño, Harold David Alvarez, Rosana Chaud, Alejandro Concepción Orozco, Gustavo Lorenzo Moretta, José Carolino Divino-

**Affiliations:** 1 Universidade Federal de Juiz de Fora, Departamento de Finanças e Controladoria e Centro de Estudos Interdisciplinares em Nefrologia (NIEPEN), Juiz de Fora, MG, Brazil.; 2 Hospital Italiano, Centro de Diálisis Peritoneal e INU Transplante, Montevideo, Uruguay.; 3 Universidade Federal de Juiz de Fora, Núcleo Interdisciplinar de Estudos e Pesquisas em Nefrologia, Juiz de Fora, MG, Brazil.; 4 Hospital de Especialidades, CMNO, IMSS, Unidad de Investigación Médica en Enfermedades Renales, Guadalajara, Mexico.; 5 Universidade Federal de Juiz de Fora, Juiz de Fora, MG, Brazil.; 6 Hospital Clínico, Caracas, Venezuela.; 7 Hospital Ramón Santamarina, Tandil, Argentina.; 8 Instituto Guatemalteco de Seguridad Social, Ciudad de Guatemala, Guatemala.; 9 Hospital de Pediatría Prof. Dr. Juan P. Garrahan, Buenos Aires, Argentina.; 10 Hospital Nacional de Maternidad Dr. Raúl Argüello Escolán y Life Center San Salvador, Hemodiálisis, San Salvador, El Salvador.; 11 Hospital Durand, Buenos Aires, Argentina.; 12 Hospital Central del Instituto de Previsión Social Dr. Emilio Cubas, Departamento de Medicina Interna, Asunción, Paraguay.; 13 Hospital Escuela Antonio Lenín Fonseca, Manágua, Nicarágua.; 14 Hospital Regional de Concepción, Concepción, Chile.; 15 Fundação Pró-Rim, Joinville, SC, Brazil.; 16 Macrotech Medical, Hospital Dr. Salvador B. Gautier, Santo Domingo, Dominican Republic.; 17 Instituto Boliviano de Nefrología, La Paz, Bolivia.; 18 Sociedad de Nefrología de Panamá, Ciudad de Panama, Panama.; 19 Hospital de Clínicas Doctor Manuel Quintela, Montevideo, Uruguay.; 20 Instituto Ecuatoriano de Seguridad Social, Quito, Ecuador.; 21 Ministerio de Salud, Lima, Peru.; 22 Universidad Nacional de Cordoba, Cordoba, Argentina.; 23 Karolinska Institutet, Stockholm, Sweden.

**Keywords:** Costs and Cost Analysis, Economics, Peritoneal Dialysis, Latin America, Delphi Technique

## Abstract

**Introduction:**

The main tools for making clinical decisions based on efficient use of resources are economic evaluation studies that allow the assessment of both the costs and benefits of different therapeutics, with appropriate guidelines for preparing reports. This study aimed to develop a checklist of consumable cost elements to be considered in estimates for micro-costing studies in peritoneal dialysis (PD).

**Methods:**

Four stages were conducted, followed by data analysis and interpretation. Three stages were carried out to develop the direct cost elements questionnaire: 1st — designing the first version of the checklist; 2nd — evaluating and expanding it using the Delphi method; 3rd — conducting two expert panels; and 4th — applying the questionnaire to professionals from 18 Latin American countries. Inclusion criteria: professionals with at least one year of clinical and/or administrative experience in PD. A discrete probability distribution adjustment was performed. Distribution lots were considered according to the category of cost elements for each country. The maximum likelihood estimation method was applied, and the statistical classification of the adjustments was assessed using the Akaike Information Criterion.

**Results:**

A total of 596 questionnaires, comprising seven dimensions and 41 elements, were validated. From the results of each batch, it was possible to segment the elements into three choice options, with the probability of evaluating an element as very important, thus allowing for the classification of the cost elements.

**Conclusion:**

The checklist favors more equitable economic dimensioning in comparative studies, making it possible to compare economic values in PD across countries, while considering the appropriate cost elements.

## Introduction

Chronic kidney disease (CKD) is a serious public health problem, affecting about 10% of the world’s population and associated with high morbidity, mortality, and costs. It is seen as a silent epidemic, constituting the eighth leading cause of death and the tenth leading cause of years of life lost^1,2,3^.

The treatment of CKD begins with primary prevention and, even if the patient’s condition progresses, it is possible to slow its progression through adequate screening, resulting in satisfactory economic effects^4,5^.

Globally, the number of people receiving chronic peritoneal dialysis (PD) is 21 pmp and increases with income level. The variation in prevalence is high in Africa: several countries report no people living with kidney disease treated with chronic PD, whereas South Africa reports a prevalence of 23.3 pmp. Hong Kong (620.8 pmp), Mexico (474 pmp), and El Salvador (380 pmp) have the highest prevalences of chronic PD, and the top three regions are North and East Asia (126 pmp), OSEA (95 pmp), and Latin America (60 pmp)^6,7^.

The impact of different dialysis reimbursement models was addressed by Piccoli et al.^8^, who differentiated the types by sessions, patients, and packages; however, this variable was also not the determining factor in choosing the type of renal replacement therapy (RRT). Thus, government spending on reimbursement for RRT services is allocated differently in a world separated by economic differences^4,5,7,9^.

In this context, in addition to the high cost, RRT is difficult to sustain from an economic point of view, even for high-income countries, and is a growing concern for health systems, as it represents a high percentage of total health expenditure^3^.

The main tools for making clinical decision based on the efficient use of resources are economic evaluation studies that allow both the costs and benefits of different therapeutic options to be estimated, with appropriate guidelines for preparing reports^10^. The quality of cost estimates is an important issue and should not be neglected, since it has the arbitrary power to change the determinants of decision-making^11,12,13^.

In 2022, when the International Society for Pharmacoeconomics and Outcomes Research (ISPOR) published the second edition of CHEERS (Consolidated Health Economic Evaluation Reporting Standards), it included in its recommendations the need to describe how costs are estimated. This encompassed describing the level of disaggregation in the identification and measurement of each consumable cost element for the provision of health services, as well as the type of approach defined (top-down versus bottom-up), considering a possible trade-off between theoretical soundness and practical feasibility^10,14^.

The top-down approach is easy to estimate and more agile, as it is generally based on financing system payment tables and corresponds to aggregated cost components, without further details. In contrast, the bottom-up approach allows for a high degree of detail and requires a checklist of consumables for the provision of health services^14,15,16^. A mixed method can also be applied, in which it is possible to consider some of the information from the top-down and bottom-up approaches as complementary, especially when complete data are not available^12,14,15,16,17^.

In general, the bottom-up approach uses micro-costing as the methodology for estimating the costs involved in health services in greater detail^14^. One criticism of micro-costing is its limitation regarding the generalization of results, which suggests the need for a checklist suitable for defining the cost elements necessary for health care^12,14^, even though there are already successful experiences of its application and standardization^15^.

In this context, a characteristic of dialysis therapies is the high level of standardization of their therapeutic protocols, which favors a more precise rationalization of the probable elements of consumption (whether goods or services) needed to provide the health service. Therefore, this study aims to develop a checklist of consumable cost elements that represent a joint effort to be considered in estimates in micro-costing studies for PD therapy.

## Methods

**Figure 1:**
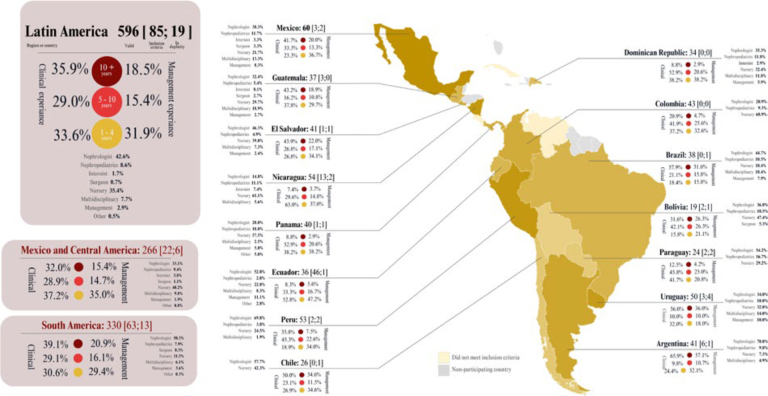
Number of professionals by country.

Four stages were used to draw up the checklist, followed by data analysis and interpretation. The first three stages were carried out to develop the questionnaire on the elements of direct costs: the first stage designed the initial version of the checklist; the second validated and improved the checklist using the Delphi method^18^; and the third conducted two panels with specialists in adult and pediatric PD. Finally, the fourth stage applied the questionnaire to professionals with experience in PD in Latin American countries (Figure 1).

### Design of the Questionnaire on Cost Elements

In the first stage, weekly online meetings were held with medical professionals who had clinical and administrative experience in the PD service, as well as researchers with experience in health economics, all of whom were participating members of the Latin American Chapter of Home Dialysis (LACDD), in order to develop a checklist of consumable cost elements using a bottom-up approach (general professionals in nephrology). Data were collected through Google Forms, which were modified as discussions progressed. The initial questionnaire indicated 22 cost elements, classified into seven groups: professionals, inputs, equipment and technology, services, patient and caregiver logistics, infrastructure, and other services (Supplementary Material).

In the second stage, the Delphi method was applied in three rounds of evaluation of the opinion questionnaire, in a spiral model, with an increase in the number of participants and countries as the technique progressed. The choice of a spiral model was based on the understanding that bringing in new evaluation experts reduced the bias in repeated assessments in each round, thereby offering the possibility of more impartial evaluations (developer specialists).

In the three rounds of the Delphi method, the experts expressed their degree of agreement with each item on the checklist using a five-level Likert scale (not important, not very important, no opinion, important, and very important). On all three occasions, the data were compiled by two medical professionals and one economics and management professional with more than 10 years of experience in PD (validator specialists).

### Characterization of the Sample and Application of the Questionnaire

In the fourth stage, the opinion questionnaire with 49 checklist items was applied to professionals from 18 Latin American countries. The request was made digitally in an electronic format, sent by email or messaging application with an access link. The opinion questionnaires were administered in Spanish and Portuguese.

As an inclusion criterion, it was decided that the professionals should have at least one year of clinical and/or administrative experience in PD services. As the population difference among the countries is large and the number of nephrologists with experience in PD is more restricted, participation was therefore limited to a maximum of 60 professionals and a minimum of 12 professionals per country, in order to avoid any overlap of opinions concentrated in countries with a greater number of available professionals, such as Argentina, Brazil, and Mexico.

**Figure 2:**
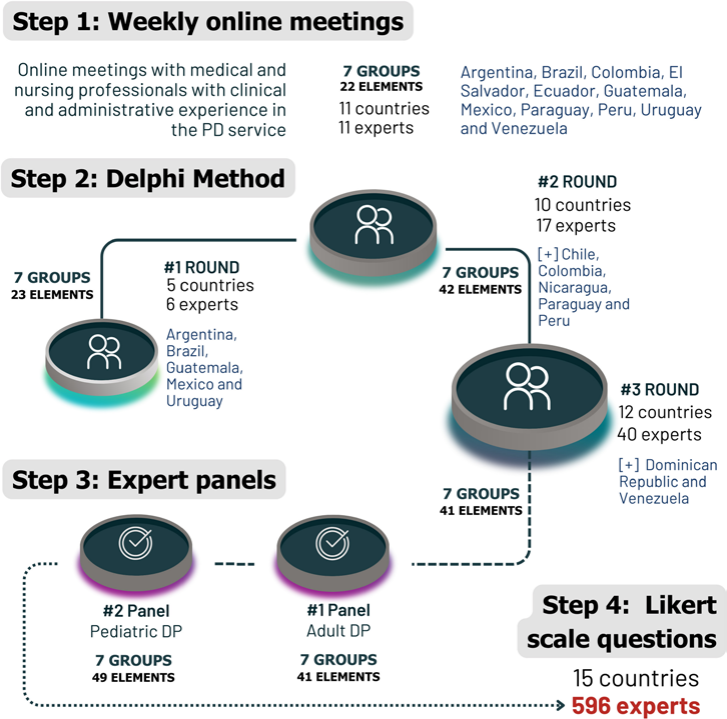
Four steps, number of participants and elements listed by the Delphi technique.

At the end of the application, 700 responses were collected, but Costa Rica, Cuba, and Venezuela did not meet the minimum participation threshold, and three responses were excluded from the sample. In addition, another 82 questionnaires were completed by professionals who did not meet the inclusion criteria and were excluded from the sample. A further 19 questionnaires were excluded because they were submitted in duplicate. Thus, 596 opinion questionnaires were validated for analysis (Figure 2).

### Ethical Considerations

The protocol was exempted from review by the Ethics Committee for Research on Human Beings under National Health Council (CNS) Resolution No. 510 of 2016, which, in Article 2, item XIV, adopts the following definition of public opinion research: *Art. 2, XIV [...] verbal or written consultation of a specific nature, carried out using a specific methodology, through which the participant is invited to express his/her preference, evaluation, or the meaning he/she attributes to themes, actions of people and organizations, or products and services, without the possibility of identifying the participant.*

### Data Analysis

To analyze the results of the qualification of the cost elements, it was applied the batch distribution adjustment for discrete data. The lots were considered by the seven groupings defined in the first stage and validated in the Delphi rounds. However, an eighth batch was formed by consumable elements specific to pediatric nephrology patients, given their specificity, and associated only with the dataset from the regions, justified by the participation of 51 pediatric nephrologists (Table 1). The 596 questionnaires were then aggregated into 18 datasets, considering each country, the surrounding regions, and a larger dataset representing Latin American (Table 2). Nevertheless, the discussion of cost elements in pediatric nephrology will not be addressed in this study.

**Table 1:** Consumable elements validated in the conception stages of the opinion questionnaire

Categories	Consumable Items	step 1	Step 2			Step 3	
		Online Meetings	Delphi Method			Expert Panel	
			1st Round	2nd Round	3rd Round	Expert nephrologists	Expert Pediatric Nephrologists
Professionals	Nephrologist	x	x	x	x	x	x
	Internist				x	x	x
	Surgeon	x	x	x	x	x	x
	Pediatric Surgeon						x
	Pediatric Nephrologist						x
	Endocrinologist						x
	Urologist						x
	Nursing	x	x	x	x	x	x
	Nutritionist	x	x	x	x	x	x
	Medical Psychology	x	x	x	x	x	x
	Social worker	x	x	x	x	x	x
Inputs (supplies)	Dialysis Bags	x	x	x	x	x	x
	Earplugs, face masks and accessories (e.g., alcohol)	x	x	x	x	x	x
	Catheter	x	x	x	x	x	x
	Drugs	x	x	x			
	Heparin				x	x	x
	Insulin				x	x	x
	Antihypertensive				x	x	x
	Antibiotic				x	x	x
	Antifungal				x	x	x
	Erythropoietin				x	x	x
	Phosphorus chelator				x	x	x
	Iron				x	x	x
	Folic acid				x	x	x
	Vitamin B				x	x	x
	Vitamin D, the stimulant				x	x	x
	Transfer Set (Transfer Line, Titanium Connector, Tweezers)	x	x	x	x	x	x
	Growth hormone						x
	Feed preparations						x
	Nasogastric tube or button						x
Equipment & Technology	Cyclers	x	x	x	x	x	x
	Telemedicine				x	x	x
	Telemonitoring					x	x
	Infusion Pump						x
Services	Catheter Implantation or Removal Services	x	x	x	x	x	x
	Hospitalization services for complications to the PD	x	x	x	x	x	x
	Paraclinical examinations	x	x	x	x	x	x
	PD training for patients and their primary care			x	x	x	x	
	Home Visit				x	x	x	
	Medical & Multidisciplinary Educational Program					x	x	
Patient & Caregiver	Patient Costs		x	x				
	Feeding				x	x	x	
	Accommodation				x	x	x	
	Tickets				x	x	x	
	Private transport costs				x	x	x	
	Transportation costs for inputs				x	x	x	
Infrastructure	Proportional Rent of Physical Space for Peritoneal Dialysis Services	x	x	x	x	x	x	
	Depreciation of own property proportional to physical space PD	x	x	x	x			
	Property maintenance costs proportional to physical space for PD services	x	x	x	x	x	x	
	Depreciation of PD Clinical Support Furniture and Equipment	x	x	x	x			
	Amortization of the initial investment				x			
	General services (e.g. telephone, water, power, internet, etc.)	x	x	x	x	x	x	
Other Professional Services	Administrative services (e.g. manager, accountant, secretary/reception, housekeeping, lawyers, information technology, etc.)	x	x	x	x	x	x	
	Ancillary maintenance services (e.g. hospital waste treatment, etc.)	x	x	x	x	x	x	

Abbreviations – PD: peritoneal dialysis; x: elements cited by respondents.

**Table 2:** Batches for probability distribution fitting

Dataset (regions/countries)	Groupings (batch fit)	n	Nephropediatrics (batch fit)	n
1. Latin America	7	596	1	51
2. Central America and Mexico	7	266	1	26
3. South America	7	330	1	25
4. Argentina	7	41		
5. Bolivia	7	19		
6. Brazil	7	38		
7. Chile	7	26		
8. Colombia	7	43		
9. Ecuador	7	36		
10. Paraguay	7	24		
11. Peru	7	53		
12. Uruguay	7	50		
13. El Salvador	7	41		
14. Guatemala	7	37		
15. Mexico	7	60		
16. Nicaragua	7	54		
17. Panama	7	40		
18. Dominican Republic	7	34		
**Total batches fit**	**126**		**3**	

A discrete sample of data based on Likert scale responses from 1 to 5 in 129 batches was considered by fitting them as distributions, in order to determine which one best represented the sample set. The maximum likelihood estimation method was applied, and the statistical classification of the adjustments was assessed using the Akaike Information Criterion (AIC) and other measures that demonstrate the absolute concentration of the binomial distribution (Table 3).

**Table 3:** Result of distribution adjustments for latin america

Cost Element Batches		Nephrologist B: Professionals				Internist B: Professionals				Surgeon B: Professionals				Infirmary B: Professionals				Nutritionist B: Professionals				Psychology B: Professionals				Social worker B: Professionals			
Distribution		Bin	InU	Then	Geo	Bin	InU	Then	Geo	Bin	InU	Then	Geo	Bin	InU	Then	Geo	Bin	InU	Then	Geo	Bin	InU	Then	Geo	Bin	InU	Then	Geo
Adjustment Statistics	AIC	◼	◻	–	.	◼	◻	–	.	◼	◻	–	.	◼	◻	–	.	◼	◻	–	.	◼	◻	–	.	◼	◻	–	.
	BIC	◼	◻	–	.	◼	◻	–	.	◼	◻	–	.	◼	◻	–	.	◼	◻	–	.	◼	◻	–	.	◼	◻	–	.
	AvLogL	◼	◻	–	.	◼	◻	–	.	◼	◻	–	.	◼	◻	–	.	◼	◻	–	.	◼	◻	–	.	◼	◻	–	.
	ChiSq	◼	–	◻	.	◼	–	◻	.	◼	–	◻	.	◼	–	◻	.	◼	◻	.	–	◼	–	◻	.	◼	–	◻	.
		Dialysis Bags B:Inputs				Personal Protective Equipment B: Inputs				Catheter B: Inputs				Heparin B: Inputs				Insulin B: Inputs				Antihypertensive B: Inputs				Antibiotics B: Inputs			
	AIC	◼	◻	–	.	◼	◻	–	.	◼	◻	–	.	◼	◻	–	.	◼	◻	–	.	◼	◻	–	.	◼	◻	–	.
	BIC	◼	◻	–	.	◼	◻	–	.	◼	◻	–	.	◼	◻	–	.	◼	◻	–	.	◼	◻	–	.	◼	◻	–	.
	AvLogL	◼	◻	–	.	◼	◻	–	.	◼	◻	–	.	◼	◻	–	.	◼	◻	–	.	◼	◻	–	.	◼	◻	–	.
	ChiSq	◼	–	◻	.	◼	–	◻	.	◼	–	◻	.	◼	–	◻	.	◼	–	◻	.	◼	–	◻	.	◼	–	◻	.
		Antifungal B:Inputs				Erythropoietin B:Inputs				Phosphorus chelator B:Inputs				Iron B:Inputs				Folic acid B:Inputs				Vitamin B B:Inputs				Vitamin D: the stimulant B: Inputs			
	AIC	◼	◻	–	.	◼	◻	–	.	◼	◻	–	.	◼	◻	–	.	◼	◻	–	.	◼	◻	–	.	◼	◻	–	.
	BIC	◼	◻	–	.	◼	◻	–	.	◼	◻	–	.	◼	◻	–	.	◼	◻	–	.	◼	◻	–	.	◼	◻	–	.
	AvLogL	◼	◻	–	.	◼	◻	–	.	◼	◻	–	.	◼	◻	–	.	◼	◻	–	.	◼	◻	–	.	◼	◻	–	.
	ChiSq	◼	–	◻	.	◼	–	◻	.	◼	–	◻	.	◼	–	◻	.	◼	–	◻	.	◼	◻	–	.	◼	–	◻	.
		Transfer Set B: Inputs				Cyclers B: Technology				Telemedicine B: Technology				Telemonitoring B: Technology				Catheter implantation or removal B: Services				Hospitalization due to complications B: Services				Paraclinical examinations B: Services			
	AIC	◼	◻	–	.	◼	◻	–	.	◼	◻	–	.	◼	◻	–	.	◼	◻	–	.	◼	◻	–	.	◼	◻	–	.
	BIC	◼	◻	–	.	◼	◻	–	.	◼	◻	–	.	◼	◻	–	.	◼	◻	–	.	◼	◻	–	.	◼	◻	–	.
	AvLogL	◼	◻	–	.	◼	◻	–	.	◼	◻	–	.	◼	◻	–	.	◼	◻	–	.	◼	◻	–	.	◼	◻	–	.
	ChiSq	◼	–	◻	.	◼	–	◻	.	◼	–	◻	.	◼	–	◻	.	◼	–	◻	.	◼	–	◻	.	◼	–	◻	.
		PD Training B: Services				Home Visit B: Services				Medical & Multidisciplinary Educational Program B: Services				Feeding B: Patient and caregiver logistics				Accommodation B: Patient and caregiver logistics				Tickets B: Patient and caregiver logistics				Private transport B: Patient and caregiver logistics			
	AIC	◼	◻	–	.	◼	◻	–	.	◼	◻	–	.	◼	◻	–	.	◼	◻	–	.	◼	◻	–	.	◼	◻	–	.
	BIC	◼	◻	–	.	◼	◻	–	.	◼	◻	–	.	◼	◻	–	.	◼	◻	–	.	◼	◻	–	.	◼	◻	–	.
	AvLogL	◼	◻	–	.	◼	◻	–	.	◼	◻	–	.	◼	◻	–	.	◼	◻	–	.	◼	◻	–	.	◼	◻	–	.
	ChiSq	◼	◻	–	.	◼	–	◻	.	◼	◻	–	.	◼	◻	–	.	◼	–	◻	.	◼	–	◻	.	◼	–	◻	.
		Transport of inputs B: Patient and caregiver logistics				Renting Physical Space B: Infrastructure				Property Maintenance B: Infrastructure				Others: telephone, water, power etc. B: Infrastructure				Management Professionals B: Other services				Other Maintenance Professionals B: Other services				Pediatric Surgeon B: Nephropediatrics			
	AIC	◼	◻	–	.	◼	◻	–	.	◼	◻	–	.	◼	◻	–	.	◼	◻	–	.	◼	◻	–	.	◼	◻	–	.
	BIC	◼	◻	–	.	◼	◻	–	.	◼	◻	–	.	◼	◻	–	.	◼	◻	–	.	◼	◻	–	.	◼	◻	–	.
	AvLogL	◼	◻	–	.	◼	◻	–	.	◼	◻	–	.	◼	◻	–	.	◼	◻	–	.	◼	◻	–	.	◼	◻	–	.
	ChiSq	◼	–	◻	.	◼	–	◻	.	◼	–	◻	.	◼	–	◻	.	◼	–	◻	.	◼	–	◻	.	◼	–	◻	.
		Pediatric Nephrologist B: Nephropediatrics				Endocrinologist B: Nephropediatrics				Urologist B:Nephropediatrics				Growth hormone B:Nephropediatrics				Food preparation B:Nephropediatrics				Nasogastric tube or button B: Nephropediatrics				Infusion Pump B: Nephropediatrics			
	AIC	◼	◻	–	.	◼	◻	–	.	◼	◻	–	.	◼	◻	–	.	◼	◻	–	.		◼			◼	◻	–	.
	BIC	◼	◻	–	.	◼	◻	–	.	◼	◻	–	.	◼	◻	–	.	◼	◻	–	.					◼	◻	–	.
	AvLogL	◼	◻	–	.	◼	◻	–	.	◼	◻	–	.	◼	◻	–	.	◼	◻	–	.					◼	◻	–	.
	ChiSq	◼	–	◻	.	◼	–	◻	.	◼	–	◻	.	◼	–	◻	.	◼	–	◻	.	◼			.	◼	–	◻	.

Goodness-of-Fit Ranking by Information Criteria (◼ #1 – AIC, ◻ #2 – BIC, – #3 – AvLogL., #4 – ChiSq)

The binomial distribution was the most appropriate. Considering its characteristic with an *n* number of samples or collections and a *p* probability of success in each attempt, the probability of success was projected for each element to be chosen as very important (Likert scale 5 in the opinion questionnaire), to the detriment of the others. Based on the results of each batch, it was possible to segment the elements into three choice options, classifying the cost elements as permanent (p ≥ 0.9), elective (0.9 > p ≥ 0.7), and unusual (p < 0.7). It is important to mention that this classification does not represent any form of exclusion of the cost element, but rather an indication of its frequency, in which those classified as unusual must receive clinical justification for their inclusion.

Understanding the possibility of some fluctuation in the choice options on the scale, we carried out a parametric bootstrap^19^ with 10,000 re-samples for each cost element, with a 95% confidence level, using the Mersenne Twister random generator^20^. The technique is widely known and is suitable for defining confidence intervals^21,22^, in which the distribution function for each of the elements was re-sampled and adjusted, determining the confidence interval estimates for the parameters. In other words, this made it feasible to deal with potential variations from the real dataset without having to make assumptions^21^. In this way, it was possible to observe the fluctuations for each country, considering their peculiarities, in which the elements may vary somewhat in their choice. All the data were calculated using @Risk 8.4 and MS Excel 2019 licensed software.

## Results

**Figure 3:**
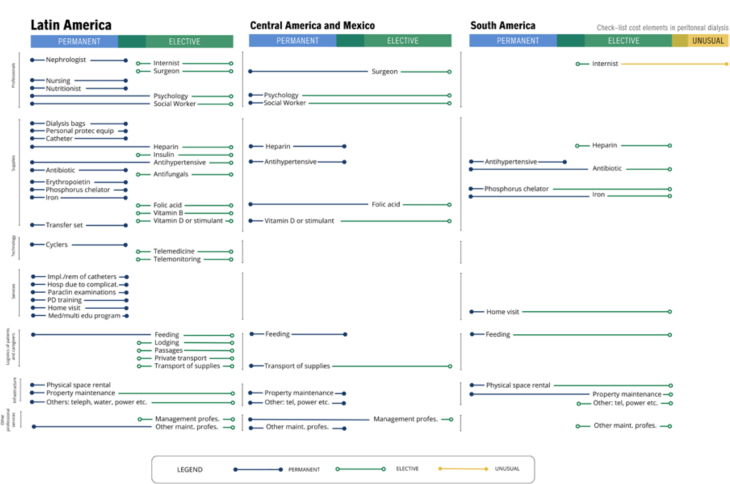
Checklist of consumables for peritoneal dialysis in studies of bottom-up micro-costing estimation for Latin America.

Figure 3 shows the seven dimensions with 41 elements, disregarding the eight elements of pediatric nephrology. In each frame, the elements are classified as “permanent”, “elective”, or “unusual”, according to the concept described above. Regarding professionals, we observed that nephrologists and nurses are considered permanent in all countries, while internists are permanent only in Nicaragua. The multidisciplinary team consisting of a social worker, nutritionist, and psychologist was not considered permanent in Chile and Paraguay. The nutritionist was not considered permanent only in Paraguay. Surgeons are considered elective in Brazil, El Salvador, Ecuador, Peru, and Uruguay.

The supplies directly related to the execution of PD were unanimously considered permanent (PD solutions, catheter, transfer set, and personal protective equipment). Then, inputs associated with PD complications, such as antibiotics, were considered permanent except in Argentina, Brazil, and Peru, while antimycotics were permanent only in Brazil, Colombia, Ecuador, Paraguay, Nicaragua, and the Dominican Republic; however, in the overall analysis of Latin America, they were considered elective. In the case of heparin, it was considered permanent in Central America and Mexico, but not in South America, despite Bolivia, Ecuador, Paraguay, and Peru classifying it as permanent.

We observed that supplies related to the treatment of CKD, such as erythropoietin, iron, and phosphorus chelators, were considered permanent in the overall Latin America analysis. Vitamin D (analogues) was considered permanent in Central America and Mexico. When analyzed separately, erythropoietin was unanimous; phosphorus chelators were not considered permanent in Paraguay and Uruguay; and iron was not considered permanent in Argentina, Paraguay, and Uruguay. Other inputs associated with comorbidities were assessed differently as elective — for example, insulin and antihypertensives in Central America and Mexico — whereas inputs associated with nutritional status, such as vitamin B and folic acid, showed variable behavior and were generally elective.

In relation to technology, PD cyclers were considered permanent, whereas the remaining items were classified as elective or unusual. Services were considered permanent throughout Latin America, except for the home visit in Uruguay. It is interesting to note that, in terms of logistics, food and transportation of inputs were considered permanent in Central America, but not in South America or in the overall Latin America. Furthermore, when analyzed separately, we observed that food was not considered permanent in Brazil and Uruguay, while in Central America and Mexico it was permanent in all countries. Transportation of inputs was also not considered permanent in Brazil and Uruguay.

Infrastructure, considering other expenses (telephone, electricity, etc.), was not considered permanent in South America. In the grouping of “other services”, management professionals were considered permanent in South America, and maintenance professionals in Central America and Mexico. In Latin America as a whole, neither of these was considered a permanent element.

## Discussion

In this study, which ultimately represented 15 Latin America countries, a checklist was drawn up with consumable cost elements in PD, which we believe will contribute to the standardization of microcosting estimates and the quality of economic studies. Recent studies have indicated the need for more consistent economic data, providing quality information in cost estimates^11,12^, and thereby reducing the production of incomplete data. In the European context, for example, there is still no consensus in the economic study guidelines regarding which costing method or approach is most appropriate for achieving the highest-quality estimates^11^. The concern with this is that the understanding of how much something costs becomes relative depending on the perspective^19^, which can influence arbitrary decisions and even generate an artificial sense of sustainable coverage of a health service^11,12,23,24.^

As expected, each country in our study presents a different payment method for the public health system, and the cost elements considered absolutely necessary, such as dialysis solutions and nurses, are similar across all countries. Several elements vary from country to country, and we will discuss each of them. We emphasize that understanding that we can evaluate the elements separately using the microcosting methodology facilitates comparison and provides more detailed information on which elements are most burdensome to the health system, thereby facilitating cost management. Thus, the checklist proposed in this study allows for a more rational understanding of the estimated economic value of health coverage, increasing the representativeness necessary for the operation of services sustainability.

PD for the treatment of CKD became widespread in the 1970s^25^. From the very beginning of the introduction of the method, the role of the nurse has been vital in carrying out different stages, from organizing the service to monitoring the patient^21^. This reflects our study’s finding that the nephrologist and the nurse are essential elements in carrying out the PD therapy, along with the patient and the family. At this point, it is worth noting that the internist is considered a permanent element in Nicaragua and elective in Mexico, Ecuador, Paraguay, and Argentina. This data is probably linked to the imbalance between the prevalence of patients on RRT and the number of nephrologists per million population, as mentioned by the SLANH in its publication on Latin America dialysis registries^6^, which means that, in these countries, there is a need for internists trained in PD. Mexico, for example, requires a nephrologist to perform HD therapy, whereas a nephrologist is not required to carry out PD therapy^25^. Moreover, in countries such as Brazil^26^, Mexico, Guatemala, Argentina, and Uruguay, there is a legal requirement for nephrologists to be part of the clinic. Some Latin America countries do not have such legislation, such as El Salvador, Paraguay, Nicaragua, and Panama. The presence of a surgeon as an effective member of the team is also not considered imperative, since in several countries nephrologists are trained to implant peritoneal catheters^27^, although in some more complex patients a surgeon may be needed.

Regarding consumables, as expected, those directly associated with the PD procedure were considered essential by all countries. Other consumables, such as those related to PD complications — antibiotics, antimycotics, and heparin — varied in importance depending on the country analyzed. It is a fact that one of the major problems is that the funder does not cover the cost of the patient’s complications, increasing the cost of PD and prolonging care time due to operational obstacles, since these drugs require a different request routine that is not part of the standard procedure. Yet they could be provided without any harm to the service, considering the pressing need for patient care and potential complications, as well as more unfavorable economic outcomes.

A study carried out in Colombia in 2017, by Makhija et al., evaluated the economic impact of a continuous quality improvement program in peritoneal dialysis and concluded that peritonitis represents a high cost for the service and that improving the quality of the service to reduce its incidence has a cost-minimizing effect^28^.

We emphasize that the use of medications directly associated with CKD treatment — such as erythropoietin, phosphorus and iron chelators, and, less frequently, vitamin D analogues — is considered essential to PD therapy. Medications associated with the treatment of comorbidities prevalent in CKD patients are not among the priorities; however, it is known that in Brazil, Nicaragua, Guatemala, El Salvador, and Panama there is access to these medications through public policies with full or partial funding.

One piece of technology that was also considered essential was the use of PD cyclers; other technologies, such as telemonitoring and telehealth, were not considered to be as important. Telemedicine has been increasingly discussed and used in the context of dialysis and showed great relevance during the COVID pandemic as an aid to managing cases virtually, without the need for face-to-face visits^29,30^. A cohort study carried out in China showed a reduction in mortality among patients telemonitored through a platform used in 26 hospitals^31^.

The “services” dimension shows that home visits have been considered essential by most countries. We know that, in the study by Martino et al., the home visit program improved the survival of PD patients and reduced the rates of peritonitis and hospitalization^32^.

The transportation of consumables is generally carried out by PD supplier companies and, therefore, there is great variation in the level of importance attributed to this information. Other infrastructure data are observed in a very variable fashion, most likely due to the limited number of studies adopting a bottom-up perspective, which considers more detailed information not available in other researches^33,34^.

Some elements are perceived as essential, such as nephrologists, nurses, PD solutions, transfer sets, PD catheters, and personal protective equipment, whereas other elements can be added according to each individual country’s vision, needs, availability, or funding conditions.

Another possibility is the comparison of the cost elements that may have been neglected (underestimated) or overestimated when compared to the amounts paid by the funding systems, which has also been observed in health economic studies^23,24,35^. These estimates make it possible to increase the transparency and detail of information for decisionmakers in a more appropriate way, offering greater reliability and flexibility for decisions in an environment with greater budgetary restrictions^36^.

However, the checklist is not intended to inhibit the selection of its cost elements; rather, it indicates the need to justify those elements that are unusual or elective. This also contributes to greater speed in defining which elements to include and flexibility in sizing options, without sacrificing the quality of the information or the need for patient care, while clearly specifying what is to be assessed, respecting both the clinical and the economical points of view.

Economic studies that use the micro-costing method reflect costs more precisely and accurately and make it possible to identify factors that are more sensitive to fluctuations in values, which in turn makes it possible to analyze cost elements with potential importance for patients and for budget definitions, as has already been demonstrated in other studies^33^.

### Limitations

Among the limitations, our study did not reach all Latin America countries; for example, Costa Rica, which has good PD penetration, did not reach the minimum number of responses required. Another point is that the study did not include a significant number of representative pediatric nephrologists per country, although it is known that there are significantly fewer of these professionals in the context of nephrology. In addition, countries such as Bolivia and Paraguay had limited representation due to low PD penetration, while in Colombia and Chile only nurses and physicians participated in the opinion poll.

As another limitation of the study, we emphasize that nephrologists from all Latin America countries were contacted, preferably those professionals who had greater access to colleagues due to their membership in organizations. Despite this, participation was voluntary and conducted through Google Forms, resulting in heterogeneity in the number of participants. However, this was balanced by defining a minimum and maximum number of respondents.

## Conclusions

We conclude, considering the reality of PD care in Latin America, that the checklist favors more isonomic economic sizing in comparative studies, making it possible to compare economic values in PD between two or more countries, considering the appropriate cost elements for each context with their respective justifications for elective and unusual elements. Despite the differences among health systems in the Latin America context, it was possible to generate a suitable checklist to support studies with more detailed information that helps to clarify PD care costs in the context of decision-making. We also highlight the contribution to decision-making that is more consistent with the representation of our reality, as well as the need to offer a PD health service with gold-standard measures of effectiveness compared with costs that have the same level of measurement quality. We therefore encourage the critical use of the checklist as an important support tool for standardizing cost descriptions, fostering a decision-making environment that is more aware of detailed, comparable, and transparent information.

## Data Availability

The datasets generated and/or analyzed during the current study are available from the corresponding author upon reasonable request.

## Supplementary Material

The following online material is available for this article:

Table S1 – Cost elements to be considered in estimates for micro-costing studies of peritoneal dialysis (PD) therapy in Latin America.
