# Erratum: Cost elements to be considered in estimates for microcosting studies in peritoneal dialysis (PD) therapy in Latin America

**DOI:** 10.1590/2175-8239-JBN-2025-0202ener

**Published:** 2026-05-18

In the article “Cost elements to be considered in estimates for microcosting studies in peritoneal dialysis (PD) therapy in Latin America”, with DOI https://doi.org/10.1590/2175-8239-JBN-2025-0202en, published in the Brazilian Journal of Nephrology, 48(2):e20250202, 2026, on page 13:

Where it reads:

We emphasize that the use of medications directly associated with CKD treatment — such as erythropoietin, phosphorus and iron chelators, and, less frequently, vitamin D analogues — is considered essential to PD therapy.

## Editorial Responsibility

Editor-in-chief: Thyago Proença de Moraes https://orcid.org/0000-0002-2983-3968.

It should read:

We emphasize that the use of medications directly associated with CKD treatment — such as erythropoietin, phosphorus and iron chelators, and, less frequently, vitamin D analogues — they are not considered essential for PD therapy.

## Editorial Responsibility

Deputy-Editor: Thyago Proença de Moraes https://orcid.org/0000-0002-2983-3968.

