# Supplementary material for: Cost elements to be considered in estimates for microcosting studies in peritoneal dialysis (PD) therapy in Latin America
Source: J Bras Nefrol. 2026 Feb 9;48(2):e20250202. doi: 10.1590/2175-8239-JBN-2025-0202en (PMC12885495; doi:10.1590/2175-8239-JBN-2025-0202en)
Supplement: Translation

# Translation: Elementos de custo a serem considerados em estimativas para estudos de microcusteio em terapia de diálise peritoneal (DP) na América Latina

**DOI:** 10.1590/2175-8239-JBN-2025-0202pt

**Authors:** Celso Souza de Moraes-, Natalia Orihuela, Natália Maria da Silva Fernandes, Laura Cortes Sanabria, Luciane Senra de Souza Braga, Pablo Amair, Uriel Winik, Angelica Viviana Manchinelli, Marta Susana Adragna, Eliseo Antonio Guzmán, Cristina Vallve, Roger Ayala Ferrari, Rodrigo José Álvarez, Hernán Jaramillo Mendoza, Viviane Calice-Silva, Erwin Iván Campos, Raul Plata-Cornejo, Regulo Valdes, Ricardo Silvariño, Harold David Alvarez, Rosana Chaud, Alejandro Concepción Orozco, Gustavo Lorenzo Moretta, José Carolino Divino-

**Affiliations:** 1 Universidade Federal de Juiz de Fora, Departamento de Finanças e Controladoria e Centro de Estudos Interdisciplinares em Nefrologia (NIEPEN), Juiz de Fora, MG, Brasil.; 2 Hospital Italiano, Centro de Diálisis Peritoneal e INU Transplante, Montevideo, Uruguay.; 3 Universidade Federal de Juiz de Fora, Núcleo Interdisciplinar de Estudos e Pesquisas em Nefrologia, Juiz de Fora, MG, Brasil.; 4 Hospital de Especialidades, CMNO, IMSS, Unidad de Investigación Médica en Enfermedades Renales, Guadalajara, Mexico.; 5 Universidade Federal de Juiz de Fora, Juiz de Fora, MG, Brasil.; 6 Hospital Clínico, Caracas, Venezuela.; 7 Hospital Ramón Santamarina, Tandil, Argentina.; 8 Instituto Guatemalteco de Seguridad Social, Ciudad de Guatemala, Guatemala.; 9 Hospital de Pediatría Prof. Dr. Juan P. Garrahan, Buenos Aires, Argentina.; 10 Hospital Nacional de Maternidad Dr. Raúl Argüello Escolán y Life Center San Salvador, Hemodiálisis, San Salvador, El Salvador.; 11 Hospital Durand, Buenos Aires, Argentina.; 12 Hospital Central del Instituto de Previsión Social Dr. Emilio Cubas, Departamento de Medicina Interna, Asunción, Paraguay.; 13 Hospital Escuela Antonio Lenín Fonseca, Manágua, Nicarágua.; 14 Hospital Regional de Concepción, Concepción, Chile.; 15 Fundação Pró-Rim, Joinville, SC, Brasil.; 16 Macrotech Medical, Hospital Dr. Salvador B. Gautier, Santo Domingo, Dominican Republic.; 17 Instituto Boliviano de Nefrología, La Paz, Bolivia.; 18 Sociedad de Nefrología de Panamá, Ciudad de Panama, Panama.; 19 Hospital de Clínicas Doctor Manuel Quintela, Montevideo, Uruguay.; 20 Instituto Ecuatoriano de Seguridad Social, Quito, Ecuador.; 21 Ministerio de Salud, Lima, Peru.; 22 Universidad Nacional de Cordoba, Cordoba, Argentina.; 23 Karolinska Institutet, Stockholm, Sweden.

**Keywords:** Custos e Análise de Custo, Economia, Diálise Peritoneal, América Latina, Técnica Delphi

## Abstract

**Introdução:**

As principais ferramentas para a tomada de decisões clínicas baseadas no uso eficiente de recursos são estudos de avaliação econômica que permitem avaliar tanto os custos quanto os benefícios de diferentes terapêuticas, com diretrizes adequadas para a elaboração de relatórios. Este estudo teve como objetivo desenvolver um *checklist* de elementos de custo de consumo a serem considerados em estimativas para estudos de microcusteio em diálise peritoneal (DP).

**Métodos:**

Foram trabalhadas quatro etapas, seguidas de análise e interpretação dos dados. Três etapas foram realizadas para o desenvolvimento do questionário de elementos de custo direto: 1ª – elaboração da primeira versão do *checklist*; 2ª – avaliação e expansão com a aplicação do método Delphi; 3ª – realização de dois painéis de especialistas; e 4ª – aplicação do questionário a profissionais de 18 países latino-americanos. Critérios de inclusão: profissionais com pelo menos um ano de experiência clínica e/ou administrativa em DP. Foi realizado um ajuste por distribuição de probabilidade discreta. Foram considerados lotes de distribuição por categoria de elementos de custo por país. Aplicou-se o método de estimação por máxima verossimilhança, e a classificação estatística dos ajustes foi estimada utilizando o Critério de Informação de Akaike.

**Resultados:**

Foram validados 596 questionários, com sete dimensões e 41 elementos. A partir dos resultados de cada lote, foi possível segmentar os elementos em três opções de escolha, com probabilidade de avaliar um elemento como muito importante, classificando assim os elementos de custo.

**Conclusão:**

O *checklist* favorece um dimensionamento econômico mais equitativo em estudos comparativos, possibilitando a comparação de valores econômicos em DP entre países, considerando os elementos de custo adequados.

## Introdução

A doença renal crônica (DRC) é um grave problema de saúde pública, afetando cerca de 10% da população mundial e sendo associada a alta morbidade, mortalidade e custos. É vista como uma epidemia silenciosa, constituindo a oitava principal causa de óbito e a décima principal causa de anos de vida perdidos^1,2,3^.

O tratamento da DRC inicia-se com a prevenção primária e, mesmo que o quadro do paciente progrida, é possível retardar sua evolução por meio de tratamento adequado, com consequentes efeitos clínicos e econômicos satisfatórios^4,5^.

Globalmente, o número de pessoas recebendo diálise peritoneal crônica (DP) é de 21 pmp e aumenta conforme o nível de renda do país. Há uma grande variação na prevalência, que é mais elevada na África, onde alguns países relatam não haver pacientes com DRC em DP, enquanto na África do Sul a prevalência é de 23,3 pmp. Hong Kong (620,8 pmp), México (474 pmp) e El Salvador (380 pmp) têm as maiores prevalências de DP crônica. No mundo, as três regiões com a maior prevalência são o Norte e o Leste da Ásia (126 pmp), OSEA (95 pmp) e América Latina (AL) (60 pmp)^6,7^.

O impacto do tipo de pagamento na penetração da DP nos países foi abordado por Picoli et al.^8^, que diferenciou o pagamento por insumos, por número de pacientes e por pacotes; entretanto, essas variáveis, isoladamente, não se revelaram determinantes na escolha do tipo de terapia renal substitutiva (TRS). Assim, o tipo de reembolso para TRS realizado pelo governo, associado ao nível econômico do país, são variáveis que determinam diferenças na penetração da DP, mas dependem do tipo de política pública, que é marcadamente diferente em um mundo separado por economias distintas^4,5,7,9^.

Nesse contexto, além do alto custo, a TRS é difícil de ser mantida pelo serviço público, do ponto de vista econômico, mesmo para países de alta renda, sendo uma preocupação crescente para os sistemas de saúde, pois representa um alto percentual do gasto total em saúde^3^.

As principais ferramentas para a tomada de decisão clínica, pautadas no uso eficiente de recursos, são os estudos de avaliação econômica, que permitem estimar tanto os custos quanto os benefícios das diferentes opções terapêuticas, com diretrizes adequadas para a elaboração dos relatórios econômicos^10^. A qualidade das estimativas de custos é uma questão importante e não deve ser negligenciada, pois tem o poder arbitrário de alterar os determinantes da tomada de decisão^11,12,13^.

Em 2022, quando a Sociedade Internacional de Farmacoeconomia e Pesquisa de Resultados (SIFPR) publicou a segunda edição do CHEERS (*Consolidated Health Economic Evaluation Reporting Standards*), foi incluída em suas recomendações a necessidade de descrever como os custos são estimados. Isso envolveu a descrição do nível de desagregação na identificação e mensuração de cada elemento de custo consumível para a prestação de serviços de saúde, bem como o tipo de abordagem definida (*top-down* versus *bottom-up*), considerando um possível *trade-off* entre solidez teórica e viabilidade prática^10,14^.

A abordagem *top-down* é fácil de estimar e mais ágil, pois geralmente se baseia em tabelas de pagamento de sistemas de financiamento e corresponde a componentes de custo agregados, sem maiores detalhes. Em contraste, a abordagem *bottom-up* permite um alto grau de detalhamento e requer uma lista de verificação de consumíveis (bens e serviços) para a prestação de serviços de saúde^14,15,16^. Um método misto também pode ser aplicado, no qual é possível considerar algumas das informações das abordagens *top-down* e *bottom-up* como complementares, especialmente quando dados completos não estão disponíveis^12,14,15,16,17^.

Em geral, a abordagem *bottom-up* escolhe os microcustos como metodologia para estimar os custos envolvidos nos serviços de saúde com maior detalhamento^14^. Uma crítica ao microcusteio é sua limitação à generalização de resultados, o que sugere a necessidade de uma lista de verificação adequada para definir os elementos de custo necessários para a assistência à saúde^12,14^, embora já existam experiências bem-sucedidas de sua aplicação e padronização^15^.

Nesse contexto, uma característica das terapias de diálise é o alto nível de padronização de seus protocolos terapêuticos, o que favorece uma racionalização mais precisa dos prováveis elementos de consumo (sejam bens ou serviços) necessários à prestação do serviço de saúde. Portanto, este estudo tem como objetivo desenvolver uma lista de verificação *(checklist*) de elementos de custo consumíveis que represente um esforço conjunto a ser considerado em estimativas em estudos de microcustos para terapia de DP.

## Métodos

Quatro etapas foram utilizadas para a elaboração do *checklist*, seguidas da análise e interpretação dos dados. As três primeiras etapas foram realizadas para o desenvolvimento do questionário sobre os elementos de custos diretos: a primeira etapa elaborou a versão inicial do *checklist*; a segunda validou e aprimorou o *checklist* utilizando o método Delphi^18^; e a terceira realizou dois painéis com especialistas em DP adulto e pediátrico. Por fim, a quarta etapa aplicou o questionário a profissionais com experiência em DP em países da AL (Figura 1).

**Figura 1:** Número de profissionais por país.

### Elaboração do Questionário sobre Elementos de Custo

Na primeira etapa, foram realizadas reuniões semanais online com profissionais médicos com experiência clínica e administrativa no serviço de DP, bem como com pesquisadores que possuem experiência em economia da saúde, todos membros participantes do Capítulo Latino-Americano de Diálise Domiciliar (LACDD), a fim de desenvolver um *checklist* de elementos de custo consumíveis com uma abordagem *bottom-up* (profissionais generalistas em nefrologia). Os dados foram coletados por meio do *Google Forms*, que foi modificado conforme o andamento das discussões. O questionário inicial indicou 22 elementos de custo, classificados em sete grupos: profissionais, insumos, equipamentos e tecnologia, serviços, logística para pacientes e cuidadores, infraestrutura e outros serviços (Material Suplementar).

Na segunda etapa, o método Delphi foi aplicado em três rodadas de avaliação do questionário de opinião, em um modelo espiral, com aumento do número de participantes e países à medida que a técnica avançava. A escolha do modelo espiral baseou-se no entendimento de que a inclusão de novos especialistas na avaliação reduzia o viés nas repetições das avaliações em cada rodada, oferecendo, assim, a possibilidade de análises mais imparciais (especialistas em desenvolvimento).

Nas três rodadas do método Delphi, os especialistas expressaram seu grau de concordância com cada item da lista de verificação usando uma escala *Likert* de cinco níveis (nada importante, pouco importante, sem opinião, importante e muito importante). Nas três ocasiões, os dados foram compilados por dois profissionais médicos e um profissional de economia e gestão, com mais de 10 anos de experiência em DP (especialistas em validação).

### Caracterização da Amostra e Aplicação do Questionário

Na quarta etapa, o questionário de opinião com 49 itens do *checklist* foi aplicado a profissionais de 18 países latino-americanos. A solicitação foi feita digitalmente, em formato eletrônico, enviada por e-mail ou aplicativo de mensagens com link de acesso. Os questionários de opinião foram aplicados em espanhol e português.

Como critério de inclusão, estabeleceu-se que os profissionais tivessem, no mínimo, um ano de experiência clínica e/ou administrativa em serviços de DP. Como a diferença populacional entre os países é grande e o número de nefrologistas com experiência em DP é mais restrito, optou-se por limitar a participação a um máximo de 60 profissionais e um mínimo de 12 profissionais por país, a fim de evitar qualquer sobreposição de opiniões concentradas em países com maior número de profissionais disponíveis, como Argentina, Brasil e México, por exemplo.

Ao final da aplicação, foram coletadas 700 respostas, mas Costa Rica, Cuba e Venezuela não atingiram o limite mínimo de participação. Três respostas foram excluídas da amostra. Além disso, outros 82 questionários foram respondidos por profissionais que não preenchiam os critérios de inclusão e foram excluídos da amostra. Observamos que 19 questionários foram enviados em duplicata e, portanto, excluídos. Assim, 596 questionários de opinião foram validados para análise (Figura 2).

**Figura 2:** Descrição das quatro etapas, número de participantes e elementos listados pela técnica Delphi.

### Considerações Éticas

O protocolo foi divulgado pelo Comitê de Ética em Pesquisa em Seres Humanos por meio da Resolução nº 510, de 2016, do Conselho Nacional de Saúde (CNS), que, em seu artigo 2º, inciso XIV, adota a seguinte definição de pesquisa de opinião pública: Art. 2.°, XIV – “[...] consulta verbal ou escrita de natureza específica, realizada com metodologia específica, por meio da qual o participante é convidado a expressar sua preferência, avaliação ou o significado que atribui a temas, ações de pessoas e organizações, ou produtos e serviços, sem possibilidade de identificação do participante.”

### Análise de Dados

Para analisar os resultados da qualificação dos elementos de custo, optou-se por aplicar o ajuste de distribuição de lotes para dados discretos. Os lotes foram considerados pelos sete agrupamentos definidos na primeira etapa e validados nas rodadas Delphi. No entanto, um oitavo lote foi formado por elementos de consumo exclusivos para pacientes de nefrologia pediátrica, dada sua especificidade, e associado apenas ao conjunto de dados das regiões, justificando-se pelo número de 51 nefrologistas pediátricos participantes (Tabela 1). Os 596 questionários foram então agregados em 18 conjuntos de dados, considerando cada país (Tabela 2). Contudo, a discussão sobre os elementos de custo em nefrologia pediátrica não será abordada em detalhes neste estudo, embora consideremos relevante manter os dados coletados em pediatria devido à paucidade de informações sobre o assunto na literatura.

**Tabela 1:** Elementos de consumo validados nas etapas de concepção do questionário de opinião

Categorias	Itens de Consumo	Fase 1	Fase 2			Fase 3	
		Reuniões Online	Método Delphi			Painel de Especialistas	
			1^a^ Rodada	2^a^ Rodada	3^a^ Rodada	Especialistas em nefrologia	Especialistas em nefrologia pediátrica
Profissionais	Nefrologista	x	x	x	x	x	x
	Internista				x	x	x
	Cirurgião	x	x	x	x	x	x
	Cirurgião Pediátrico						x
	Nefrologista Pediátrico						x
	Endocrinologista						x
	Urologista						x
	Enfermagem	x	x	x	x	x	x
	Nutricionista	x	x	x	x	x	x
	Psicologia Médica	x	x	x	x	x	x
	Assistente Social	x	x	x	x	x	x
	Bolsas de Diálise	x	x	x	x	x	x
Insumos (suprimentos)	Protetores auriculares, máscaras faciais e acessórios (por exemplo, álcool)	x	x	x	x	x	x
	Cateter	x	x	x	x	x	x
	Medicamentos	x	x	x			
	Heparina				x	x	x
	Insulina				x	x	x
	Anti-hipertensivos				x	x	x
	Antibióticos				x	x	x
	Antifúngicos				x	x	x
	Eritropoietina				x	x	x
	Quelante de fósforo				x	x	x
	Ferro				x	x	x
	Ácido fólico				x	x	x
	Vitamina B				x	x	x
	Vitamina D, estimulante				x	x	x
	Conjunto de transferência (linha de transferência, conector de titânio, pinça)	x	x	x	x	x	x
	Hormônio do crescimento						x
	Preparo de alimentos						x
	Sonda ou *botton* de gastrostomia						x
Equipamentos & Tecnologia	Cicladores	x	x	x	x	x	x
	Telemedicina				x	x	x
	Telemonitoramento					x	x
	Bomba de infusão						x
Serviços	Serviços de implantação ou remoção de cateter	x	x	x	x	x	x
	Serviços de hospitalização para complicações relacionadas à DP	x	x	x	x	x	x
	Exames paraclínicos	x	x	x	x	x	x
	Treinamento em DP para pacientes e seus cuidadores principais			x	x	x	x
	Visita domiciliar				x	x	x
	Programa Educacional Médico e Multiprofissional					x	x
Paciente & Cuidador	Custos do Paciente		x	x			
	Alimentação				x	x	x
	Hospedagem				x	x	x
	Passagens				x	x	x
	Custos de transporte privado				x	x	x
	Custos de transporte de insumos				x	x	x
Infraestrutura	Aluguel proporcional do espaço físico destinado aos serviços de diálise peritoneal	x	x	x	x	x	x
	Depreciação do imóvel próprio proporcional ao espaço físico dedicado à DP	x	x	x	x		
	Custos de manutenção do imóvel proporcionais ao espaço físico destinado aos serviços de DP	x	x	x	x	x	x
	Depreciação do mobiliário e equipamentos de apoio clínico à DP	x	x	x	x		
	Amortização do investimento inicial				x		
	Serviços gerais (por exemplo, telefone, água, energia elétrica, internet etc.)	x	x	x	x	x	x
Outros Serviços Profissionais	Serviços administrativos (por exemplo, gerente, contador, secretaria/recepção, limpeza, advogados, tecnologia da informação etc.)	x	x	x	x	x	x
	Serviços auxiliares de manutenção (por exemplo, tratamento de resíduos hospitalares etc.)	x	x	x	x	x	x

Abreviaturas – DP: diálise peritoneal; x: elementos citados pelos respondentes.

**Tabela 2:** Lotes para ajuste da distribuição de probabilidade

Conjunto de dados (regiões/países)	Agrupamentos (ajuste por lote)	n	Nefropediatria (ajuste por lote)	n
1. América Latina	7	596	1	51
2. América Central e México	7	266	1	26
3. América do Sul	7	330	1	25
4. Argentina	7	41		
5. Bolívia	7	19		
6. Brasil	7	38		
7. Chile	7	26		
8. Colômbia	7	43		
9. Equador	7	36		
10. Paraguai	7	24		
11. Peru	7	53		
12. Uruguai	7	50		
13. El Salvador	7	41		
14. Guatemala	7	37		
15. México	7	60		
16. Nicarágua	7	54		
17. Panamá	7	40		
18. República Dominicana	7	34		
**Total de lotes ajustados**	**126**		**3**

Uma amostra discreta de dados baseada em respostas em escala *Likert* de 1 a 5 em 129 lotes foi considerada, ajustando-as como distribuições, a fim de determinar qual delas melhor representava o conjunto amostral. Foi aplicado o método de Estimativa de Máxima Verossimilhança, e a classificação estatística dos ajustes foi estimada utilizando o Critério de Informação de Akaike (AIC) e outros ajustes que demonstram a concentração absoluta da distribuição binomial (Tabela 3).

**Tabela 3:** Resultado dos ajustes de distribuição para a américa latina

Elemento de custo Lotes		Nefrologista B: Profissionais				Internista B: Profissionais				Cirurgião B: Profissionais				Enfermaria B: Profissionais				Nutricionista B: Profissionais				Psicologia B: Profissionais				Assistente social B: Profissionais			
Distribuição		Bin	InU	Then	Geo	Bin	InU	Then	Geo	Bin	InU	Then	Geo	Bin	InU	Then	Geo	Bin	InU	Then	Geo	Bin	InU	Then	Geo	Bin	InU	Then	Geo
Estatísticas de Ajuste	AIC	◼	◻	–	.	◼	◻	–	.	◼	◻	–	.	◼	◻	–	.	◼	◻	–	.	◼	◻	–	.	◼	◻	–	.
	BIC	◼	◻	–	.	◼	◻	–	.	◼	◻	–	.	◼	◻	–	.	◼	◻	–	.	◼	◻	–	.	◼	◻	–	.
	AvLogL	◼	◻	–	.	◼	◻	–	.	◼	◻	–	.	◼	◻	–	.	◼	◻	–	.	◼	◻	–	.	◼	◻	–	.
	ChiSq	◼	–	◻	.	◼	–	◻	.	◼	–	◻	.	◼	–	◻	.	◼	◻	.	–	◼	–	◻	.	◼	–	◻	.
		Bolsas de diálise B:Insumos				Equipamento de Proteção Individual (EPI) B: Insumos				Cateter B: Insumos				Heparina B: Insumos				Insulina B: Insumos				Anti-hipertensivo B: Insumos				Antibióticos B: Insumos			
	AIC	◼	◻	–	.	◼	◻	–	.	◼	◻	–	.	◼	◻	–	.	◼	◻	–	.	◼	◻	–	.	◼	◻	–	.
	BIC	◼	◻	–	.	◼	◻	–	.	◼	◻	–	.	◼	◻	–	.	◼	◻	–	.	◼	◻	–	.	◼	◻	–	.
	AvLogL	◼	◻	–	.	◼	◻	–	.	◼	◻	–	.	◼	◻	–	.	◼	◻	–	.	◼	◻	–	.	◼	◻	–	.
	ChiSq	◼	–	◻	.	◼	–	◻	.	◼	–	◻	.	◼	–	◻	.	◼	–	◻	.	◼	–	◻	.	◼	–	◻	.
		Antifúngicos B: Insumos				Eritropoietina B: Insumos				Quelante de fósforo B: Insumos				Ferro B: Insumos				Ácido fólico B: Insumos				Vitamina B B: Insumos				Vitamina D: estimulante B: Insumos			
	AIC	◼	◻	–	.	◼	◻	–	.	◼	◻	–	.	◼	◻	–	.	◼	◻	–	.	◼	◻	–	.	◼	◻	–	.
	BIC	◼	◻	–	.	◼	◻	–	.	◼	◻	–	.	◼	◻	–	.	◼	◻	–	.	◼	◻	–	.	◼	◻	–	.
	AvLogL	◼	◻	–	.	◼	◻	–	.	◼	◻	–	.	◼	◻	–	.	◼	◻	–	.	◼	◻	–	.	◼	◻	–	.
	ChiSq	◼	–	◻	.	◼	–	◻	.	◼	–	◻	.	◼	–	◻	.	◼	–	◻	.	◼	◻	–	.	◼	–	◻	.
		Conjunto de Transferência B: Insumos				Cicladores B: Tecnologia				Telemedicina B: Tecnologia				Telemonitoramento B: Tecnologia				Implantação ou remoção de cateter B: Serviços				Hospitalização devido a complicações B: Serviços				Exames paraclínicos B: Serviços			
	AIC	◼	◻	–	.	◼	◻	–	.	◼	◻	–	.	◼	◻	–	.	◼	◻	–	.	◼	◻	–	.	◼	◻	–	.
	BIC	◼	◻	–	.	◼	◻	–	.	◼	◻	–	.	◼	◻	–	.	◼	◻	–	.	◼	◻	–	.	◼	◻	–	.
	AvLogL	◼	◻	–	.	◼	◻	–	.	◼	◻	–	.	◼	◻	–	.	◼	◻	–	.	◼	◻	–	.	◼	◻	–	.
	ChiSq	◼	–	◻	.	◼	–	◻	.	◼	–	◻	.	◼	–	◻	.	◼	–	◻	.	◼	–	◻	.	◼	–	◻	.
		Treinamento em DP B: Serviços				Visita Domiciliar B: Serviços				Programa Educacional Médico e Multiprofissional B: Serviços				Alimentação B: Logística para pacientes e cuidadores				Hospedagem B: Logística para pacientes e cuidadores				Passagens B: Logística para pacientes e cuidadores				Transporte privado B: Logística para pacientes e cuidadores			
	AIC	◼	◻	–	.	◼	◻	–	.	◼	◻	–	.	◼	◻	–	.	◼	◻	–	.	◼	◻	–	.	◼	◻	–	.
	BIC	◼	◻	–	.	◼	◻	–	.	◼	◻	–	.	◼	◻	–	.	◼	◻	–	.	◼	◻	–	.	◼	◻	–	.
	AvLogL	◼	◻	–	.	◼	◻	–	.	◼	◻	–	.	◼	◻	–	.	◼	◻	–	.	◼	◻	–	.	◼	◻	–	.
	ChiSq	◼	◻	–	.	◼	–	◻	.	◼	◻	–	.	◼	◻	–	.	◼	–	◻	.	◼	–	◻	.	◼	–	◻	.
		Transporte de insumos B: Logística para pacientes e cuidadores				Aluguel de espaço físico B: Infraestrutura				Manutenção do imóvel B: Infraestrutura				Outros: telefone, água, energia elétrica etc. B: Infraestrutura				Profissionais de gestão B: Outros serviços				Outros profissionais de manutenção B: Outros serviços				Cirurgião Pediátrico B: Nefropediatria			
	AIC	◼	◻	–	.	◼	◻	–	.	◼	◻	–	.	◼	◻	–	.	◼	◻	–	.	◼	◻	–	.	◼	◻	–	.
	BIC	◼	◻	–	.	◼	◻	–	.	◼	◻	–	.	◼	◻	–	.	◼	◻	–	.	◼	◻	–	.	◼	◻	–	.
	AvLogL	◼	◻	–	.	◼	◻	–	.	◼	◻	–	.	◼	◻	–	.	◼	◻	–	.	◼	◻	–	.	◼	◻	–	.
	ChiSq	◼	–	◻	.	◼	–	◻	.	◼	–	◻	.	◼	–	◻	.	◼	–	◻	.	◼	–	◻	.	◼	–	◻	.
		Nefrologista Pediátrico B: Nefropediatria				Endocrinologista B: Nefropediatria				Urologista B: Nefropediatria				Hormônio do crescimento B: Nefropediatria				Preparo de alimentos B: Nefropediatria				Sonda ou de gastrostomia B: Nefropediatria				Bomba de infusão B: Nefropediatria			
	AIC	◼	◻	–	.	◼	◻	–	.	◼	◻	–	.	◼	◻	–	.	◼	◻	–	.		◼			◼	◻	–	.
	BIC	◼	◻	–	.	◼	◻	–	.	◼	◻	–	.	◼	◻	–	.	◼	◻	–	.					◼	◻	–	.
	AvLogL	◼	◻	–	.	◼	◻	–	.	◼	◻	–	.	◼	◻	–	.	◼	◻	–	.					◼	◻	–	.
	ChiSq	◼	–	◻	.	◼	–	◻	.	◼	–	◻	.	◼	–	◻	.	◼	–	◻	.	◼			.	◼	–	◻	.

Notas – Classificação da Qualidade do Ajuste (*goodness-of-fit*) por Critérios de Informação (◼ #1 – AIC, ◻ #2 – BIC, – #3 – AvLogL., #4 – ChiSq). Bin: Binomial; InU: Uniforme inteira; Then: Triangular; Geo: Geométrica.

A distribuição binomial mostrou-se mais adequada. Considerando sua característica com *n* número de amostras ou coletas e *p* probabilidades de sucesso em cada tentativa, projetou-se a probabilidade de sucesso para que cada elemento fosse escolhido como muito importante (escala *Likert* 5 no questionário de opinião), em detrimento dos demais. Com base nos resultados de cada lote, foi possível segmentar os elementos em três opções de escolha, classificando-os em: permanente (p ≥ 0,9), eletivo (0,9 > p ≥ 0,7) e incomum (p < 0,7). É importante mencionar que essa classificação não representa qualquer tipo de exclusão do elemento de custo, mas sim uma indicação de sua frequência, na qual aqueles que são incomuns devem receber justificativa clínica para sua inclusão.

Compreendendo a possibilidade de alguma flutuação nas opções de escolha na escala, realizamos um *bootstrap* paramétrico^19^ com 10.000 reamostragens para cada elemento de custo, com um nível de confiança de 95%, usando o gerador aleatório *Mersenne Twister* ^20^. A técnica é amplamente conhecida e adequada para definir intervalos de confiança^21,22^, nos quais a função de distribuição para cada um dos elementos foi reamostrada e ajustada, determinando as estimativas do intervalo de confiança para os parâmetros. Em outras palavras, isso tornou possível lidar com possíveis variações do conjunto de dados real sem a necessidade de fazer suposições^21^. Dessa forma, foi possível observar as flutuações para cada país, levando em consideração suas peculiaridades, nas quais os elementos podem apresentar pequenas variações em sua escolha. Todos os dados foram calculados utilizando o software licenciado @Risk 8.4 e MS Excel 2019.

## Resultados

A Figura 3 apresenta as sete dimensões com 41 elementos, desconsiderando os oito elementos da nefrologia pediátrica. Em cada quadro, são apresentados os elementos “permanentes”, “eletivos” ou “incomuns”, de acordo com o conceito descrito acima. Em relação aos profissionais, observamos que nefrologistas e enfermeiros são considerados permanentes em todos os países, enquanto os clínicos gerais são permanentes apenas na Nicarágua. A equipe multiprofissional composta por assistente social, nutricionista e psicólogo não foi considerada permanente no Chile e no Paraguai. O nutricionista não foi considerado permanente apenas no Paraguai. Cirurgiões são considerados eletivos no Brasil, El Salvador, Equador, Peru e Uruguai.

**Figura 3:** Lista de materiais de consumo para diálise peritoneal em estudos de estimativa de microcustos *bottom-up* para a América Latina.

Os insumos diretamente relacionados à execução da DP foram unanimemente considerados permanentes (soluções para DP, cateter, conjunto de transferência e equipamentos de proteção individual). Em seguida, os insumos associados às complicações da DP, como antibióticos, foram considerados permanentes, exceto na Argentina, Brasil e Peru. Enquanto os antimicóticos foram considerados permanentes apenas no Brasil, Colômbia, Equador, Paraguai, Nicarágua e República Dominicana, na análise global da AL, eles foram classificados como eletivos. No caso da heparina, o insumo foi considerado permanente na América Central e no México, mas não em todos os países da América do Sul, embora Bolívia, Equador, Paraguai e Peru a tenham considerado permanente.

Observamos que os insumos relacionados ao tratamento da DRC, como eritropoietina, quelantes de ferro e fósforo, foram considerados permanentes na AL em geral. A vitamina D (análogos) foi considerada permanente na América Central e no México. Quando analisados separadamente, a eritropoietina foi unânime; os quelantes de fósforo não foram considerados permanentes no Paraguai e no Uruguai; e o ferro não foi considerado permanente na Argentina, Paraguai e Uruguai. Outros insumos associados a comorbidades foram avaliados de forma diferente, como eletivos — por exemplo, insulina e anti-hipertensivos na América Central e no México — enquanto os insumos associados ao estado nutricional, como vitamina B e ácido fólico, apresentaram comportamento variável e foram geralmente eletivos.

Em relação à tecnologia, a máquina de DP automatizada foi considerada permanente, enquanto os demais itens foram classificados como eletivos ou incomuns. Os serviços foram considerados permanentes em toda a AL, exceto a visita domiciliar no Uruguai. É interessante notar que, em termos de logística, alimentação e transporte de insumos foram considerados permanentes na América Central, mas não na América do Sul, nem quando se considera a AL como um todo. Ainda na análise por país, observamos que a alimentação não foi considerada permanente no Brasil e no Uruguai, enquanto na América Central e no México foi classificada como permanente. O transporte de insumos também não foi considerado permanente no Brasil e no Uruguai.

A infraestrutura, considerando outras despesas (telefone, energia elétrica etc.), não foi considerada permanente na América do Sul. No agrupamento de “outros serviços”, profissionais de gestão foram classificados como permanentes na América do Sul, enquanto profissionais de manutenção foram considerados permanentes na América Central e no México. Na AL como um todo, ambos não foram considerados como elementos permanentes.

## Discussão

Neste estudo, que representou 15 países da AL, elaborou-se um *checklist* com elementos de custo consumíveis em DP, o que acreditamos contribuirá para a padronização das estimativas de microcusteio e para a qualidade dos estudos econômicos. Estudos recentes têm indicado a necessidade de dados econômicos mais consistentes, fornecendo informações de qualidade nas estimativas de custo^11,12^ e, assim, reduzindo a produção de dados incompletos. No contexto europeu, por exemplo, ainda não há consenso, nas diretrizes de estudos econômicos, sobre qual método ou abordagem de custeio é mais apropriado para estimativas de altíssima qualidade^11^. A preocupação com isso é que a compreensão de quanto custa se torne relativa dependendo da perspectiva^19^, o que pode influenciar decisões arbitrárias e até mesmo gerar uma sensação artificial de cobertura sustentável de um serviço de saúde^11,12,23,24^.

Como esperado, cada país do nosso estudo apresenta uma forma de pagamento diferente para o sistema público de saúde, e os elementos de custo considerados absolutamente necessários, como soluções de diálise e enfermeiros, são semelhantes em todos os países. Diversos elementos variam de país para país, e cada um deles será discutido. Ressaltamos que a compreensão de que é possível avaliar os elementos separadamente utilizando a metodologia de microcusteio facilita a comparação e fornece informações mais detalhadas sobre quais elementos são mais onerosos para o sistema de saúde, facilitando a gestão de custos. Assim, o *checklist* proposto neste estudo permite uma compreensão mais racional do valor econômico estimado da cobertura de saúde, aumentando o poder de representação necessário para a operação da sustentabilidade dos serviços.

A DP para o tratamento da DRC se generalizou na década de 1970^25^. Desde o início da introdução do método, o papel do enfermeiro tem sido vital na execução de diferentes etapas, desde a organização do serviço até o acompanhamento do paciente^21^. Isso se reflete na constatação do nosso estudo de que o nefrologista e o enfermeiro são elementos essenciais na execução da terapia de DP, juntamente com o paciente e a família. Nesse ponto, vale destacar que o clínico geral é considerado um elemento permanente na Nicarágua e eletivo no México, Equador, Paraguai e Argentina. Esses dados provavelmente estão relacionados ao desequilíbrio entre a prevalência de pacientes em TRS e o número de nefrologistas por milhão de habitantes, conforme mencionado pela Sociedade Latino-Americana de Nefrologia e Hipertensão (SLANH) em sua publicação sobre registros de diálise na AL^6^, o que indica que, nesses países, há necessidade de médicos internistas treinados em DP. O México, por exemplo, exige um nefrologista para realizar a terapia de HD, enquanto um nefrologista não é necessário para realizar a terapia de DP^25^. Além disso, em países como Brasil^26^, México, Guatemala, Argentina e Uruguai, há exigência legal para que nefrologistas façam parte da clínica. Alguns países da AL não possuem tal legislação, como El Salvador, Paraguai, Nicarágua e Panamá. A presença de um cirurgião como membro efetivo da equipe também não é considerada imperativa, visto que, em vários países, os nefrologistas são treinados para implantar cateteres peritoneais^27^, embora em alguns pacientes mais complexos um cirurgião possa ser necessário.

Em relação aos consumíveis, como esperado, aqueles diretamente associados ao procedimento de DP foram considerados essenciais por todos os países. Outros consumíveis, como aqueles relacionados às complicações da DP — uso de antibióticos, antimicóticos e heparina — variaram em importância dependendo do país analisado. É fato que um dos principais problemas é que o financiador não cobre o custo das complicações do paciente, o que aumenta o custo da DP e prolonga o tempo de atendimento devido a possíveis obstáculos operacionais, uma vez que esses medicamentos exigem uma rotina diferenciada para solicitações que não fazem parte do procedimento padrão, enquanto poderiam ser providenciados sem prejuízo ao serviço, considerando a necessidade urgente de atendimento ao paciente, as possíveis complicações e os desfechos econômicos mais desfavoráveis.

Um estudo realizado na Colômbia em 2017, por Makhija et al., avaliou o impacto econômico de um programa de melhoria contínua da qualidade em DP e concluiu que a peritonite representa um alto custo para o serviço e que melhorar a qualidade do serviço para reduzir sua incidência tem um efeito de minimização de custos^28^.

Ressaltamos que o uso de medicamentos diretamente associados ao tratamento da DRC, como eritropoietina, quelantes de fósforo e ferro, e, menos frequentemente, análogos da vitamina D, é considerado essencial para a terapia de DP. Medicamentos associados ao tratamento de comorbidades prevalentes em pacientes com DRC não estão entre as prioridades; é sabido que, no Brasil, Nicarágua, Guatemala, El Salvador e Panamá, há acesso a esses medicamentos por meio de políticas públicas com financiamento total ou parcial.

Uma tecnologia que também foi considerada essencial foi o uso das máquinas de diálise peritoneal automatizada, ou cicladoras; outras tecnologias, como telemonitoramento e telessaúde, não foram consideradas tão importantes. A telemedicina tem sido cada vez mais discutida e utilizada no contexto da diálise e demonstrou grande relevância durante a pandemia de COVID-19 como auxílio ao manejo de casos de forma virtual, sem a necessidade de consultas presenciais^29,30^. Um estudo de coorte realizado na China mostrou redução da mortalidade em pacientes telemonitorados por meio de uma plataforma utilizada em 26 hospitais^31^.

A dimensão “serviços” mostra que as visitas domiciliares têm sido consideradas essenciais pela maioria dos países. Sabe-se que, no estudo de Martino et al., o programa de visitas domiciliares melhorou a sobrevida de pacientes em DP, bem como reduziu as taxas de peritonite e de hospitalização^32^.

O transporte de consumíveis geralmente é realizado por empresas fornecedoras de DP e, portanto, há grande variação no nível de importância atribuído a essas informações. Outros dados de infraestrutura são observados de forma bastante variável, provavelmente devido aos poucos estudos com perspectiva *bottom-up*, que consideram informações mais detalhadas; portanto, essas informações não estão disponíveis em outros estudos^33,34^.

Alguns elementos são percebidos como essenciais, como nefrologistas, enfermeiros, soluções de DP, conjunto de transferência, cateteres de DP e equipamentos de proteção individual, enquanto outros podem ser adicionados de acordo com a visão, as necessidades, a disponibilidade ou as condições de financiamento de cada país.

Outra possibilidade é a comparação dos elementos de custo que podem ter sido negligenciados (subestimados) ou superestimados em relação aos valores pagos pelos sistemas de financiamento, o que também foi observado em estudos de economia da saúde^23,24,35^. Essas estimativas permitirão aumentar a transparência e o detalhamento das informações para os tomadores de decisão de forma mais adequada, oferecendo maior confiabilidade e flexibilidade para as decisões em um ambiente de maiores restrições orçamentárias^36^.

No entanto, essa lista de verificação não tem a intenção de inibir a seleção de seus elementos de custo, mas sim de indicar a necessidade de justificar aqueles que são incomuns ou eletivos, enquanto os permanentes são considerados essenciais. Isso também contribui para maior rapidez na definição dos elementos a serem incluídos e para maior flexibilidade no dimensionamento das opções, sem renunciar à qualidade da informação e à necessidade de cuidado do paciente, mantendo claro o que será avaliado e respeitando o ponto de vista clínico e econômico.

Estudos econômicos que utilizam o método de microcusteio refletem os custos de forma mais precisa e acurada e permitem identificar fatores mais sensíveis às flutuações de valores, o que possibilita a análise de elementos de custo com potencial importância para o paciente e para a definição do orçamento, como já demonstrado em outros estudos^33^.

### Limitações

Entre as limitações, nosso estudo não abrangeu todos os países da AL. Por exemplo, a Costa Rica, que possui uma boa penetração em DP, não atingiu o número mínimo de respostas necessário. Outro ponto é que o estudo não incluiu um número significativo de nefrologistas pediátricos representativos por país, embora saibamos que há um número significativamente menor desses profissionais no contexto da nefrologia. Além disso, países como Bolívia e Paraguai tiveram pouca representatividade devido à baixa penetração em DP, enquanto Colômbia e Chile tiveram apenas enfermeiros e médicos participando da pesquisa de opinião.

Como outra limitação do estudo, destacamos que nefrologistas de todos os países da AL foram contatados, preferencialmente aqueles profissionais que tinham maior acesso a colegas devido à sua filiação a organizações. Apesar disso, a participação foi voluntária e realizada por meio do *Google Forms*, havendo heterogeneidade no número de participantes. No entanto, isso foi equilibrado pela definição de um número mínimo e máximo de participantes.

## Conclusões

Concluímos, considerando a realidade do cuidado em DP na AL, que o *checklist* favorece um dimensionamento econômico mais isonômico em estudos comparativos, possibilitando a comparação de valores econômicos em DP entre dois ou mais países, levando em conta os elementos de custo adequados para cada contexto, com suas respectivas justificativas para elementos eletivos e incomuns. Apesar das diferenças entre os sistemas de saúde no contexto da AL, foi possível gerar um *checklist* adequado para subsidiar estudos com informações mais detalhadas, que auxiliem na compreensão dos custos do cuidado em DP no processo da tomada de decisão. Destacamos também a contribuição para uma tomada de decisão mais condizente com a representação da nossa realidade, bem como a necessidade de oferecer um serviço de saúde em DP com medidas de efetividade padrão-ouro, em comparação a custos que também apresentem o mesmo nível de qualidade de mensuração. Portanto, incentivamos o uso crítico do *checklist* como importante ferramenta de apoio à padronização das descrições de custos, favorecendo um ambiente de tomada de decisão mais atento a informações detalhadas, comparáveis e transparentes.

## Data Availability

Os conjuntos de dados gerados e/ou analisados durante o estudo atual estão disponíveis com o autor correspondente mediante solicitação.

## Material Suplementar

O seguinte material online está disponível para o presente artigo:

Tabela S1 – Elementos de custo a serem considerados em estimativas para estudos de< microcusteio em terapia de diálise peritoneal (DP) na América Latina.
