# Supplementary material for: Erratum: Cost elements to be considered in estimates for microcosting studies in peritoneal dialysis (PD) therapy in Latin America
Source: J Bras Nefrol. 2026 May 18;48(2):e20250202er. doi: 10.1590/2175-8239-JBN-2025-0202ener (PMC13183358; doi:10.1590/2175-8239-JBN-2025-0202ener)
Supplement: Related texts

# Correction: Errata: Elementos de custo a serem considerados em estimativas para estudos de microcusteio em terapia de diálise peritoneal (DP) na América Latina

**DOI:** 10.1590/2175-8239-JBN-2025-0202pter

No artigo “Elementos de custo a serem considerados em estimativas para estudos de microcusteio em terapia de diálise peritoneal (DP) na América Latina”, com o DOI https://doi.org/10.1590/2175-8239-JBN-2025-0202pt, publicado no periódico Brazilian Journal of Nephrology, 48(2):e20250202, 2026, na página 13:

Onde lê-se:

Ressaltamos que o uso de medicamentos diretamente associados ao tratamento da DRC, como eritropoietina, quelantes de fósforo e ferro, e, menos frequentemente, análogos da vitamina D, é considerado essencial para a terapia de DP.

## Responsabilidade Editorial

Editor-chefe: Thyago Proença de Moraes https://orcid.org/0000-0002-2983-3968.

Leia-se:

Ressaltamos que o uso de medicamentos diretamente associados ao tratamento da DRC, como eritropoietina, quelantes de fósforo e ferro, e, menos frequentemente, análogos da vitamina D, não são considerados essenciais para a terapia de DP.

## Responsabilidade Editorial

Vice-Editor: Thyago Proença de Moraes https://orcid.org/0000-0002-2983-3968.
